# Evaluation of Presumptive Normal Feline Tonsils with Low-Field Magnetic Resonance Imaging: A Preliminary Retrospective Study

**DOI:** 10.3390/vetsci10100619

**Published:** 2023-10-14

**Authors:** Martina Rosto, Francesca Del Signore, Nicola Bernabò, Andrea De Bonis, Sara Canal, Andrea Paolini, Roberto Tamburro, Amanda Bianchi, Massimo Vignoli

**Affiliations:** 1 Department of Veterinary Medicine, University of Teramo, 64100 Località Piano d’Accio, Italy; fdelsignore@unite.it (F.D.S.); adebonis@unite.it (A.D.B.); scanal@unite.it (S.C.); apaolini@unite.it (A.P.); rtamburro@unite.it (R.T.); abianchi@unite.it (A.B.); 2 Department of Bioscience and Technology for Food, Agriculture and Environment, University of Teramo, Via Renato Balzarini 1, 64100 Teramo, Italy; nbernabo@unite.it

**Keywords:** feline palatine tonsils, low-field MRI, healthy cats

## Abstract

**Simple Summary:**

Palatine tonsils are lymphoid organs within the tonsillar fossa. Their location provides them a role against antigens entering the body during feeding and breathing. MRI is considered the technique of choice in depicting soft tissue, and it can show concurrent bone involvement. In human medicine, MRI is used in the investigation of tonsillar diseases. In veterinary medicine, a recent study on healthy dogs has described the MRI appearance of presumed normal canine palatine tonsils. Due to the similarities between animals and humans, the authors aimed to evaluate the feasibility of low-field MRI to detect tonsils in cats and to describe features of presumed normal palatine tonsils, assessing the interoperator reproducibility of the findings in a population of adult cats. MRI is a potentially useful imaging modality for the assessment of palatine tonsils in cats; also, with a low-field MRI, their normal appearance has been described for the first time in this study. The authors recommend the evaluation of tonsils in the transverse plane and consider the most accurate estimation of the short axis. This study could be a baseline for the evaluation of MRI in the assessment of feline tonsils with high-field MRI.

**Abstract:**

Palatine tonsils are lymphoid organs, whose anatomic localization gives them a role against antigens entering the body during feeding and breathing. In human medicine, MRI is used to investigate tonsillar diseases. In veterinary medicine, a recent study on healthy dogs described the MRI appearance of canine palatine tonsils, with no available reports about feline ones. Due to the similarities between animals and humans, and based on the study on canine tonsils, the authors aimed to evaluate the feasibility of low-field MRI to detect and describe presumed normal features of feline palatine tonsils, assessing the finding’s reproducibility. Low-field MRI of the heads of 14 cats was reviewed, and qualitative findings (visualization, shape, margins, signal intensity, and pattern) and size of each tonsil were recorded. Each observer recorded 71% of the expected tonsils. Most of them were classified as oval, ill-defined, and hyperintense structures with both homogeneous and heterogeneous signal patterns; the overall agreement was considered good. Low-field MRI is potentially a useful imaging modality to visualize palatine tonsils in cats, and their normal appearance has been described for the first time. The authors recommend the evaluation of tonsils in the transverse plane and consider the most accurate estimation of the short axis.

## 1. Introduction

Several tonsils are present in cats depending on their anatomical location, including the lingual tonsil, the paired palatine tonsil, the paired para epiglottic tonsil, and the pharyngeal tonsil. The lingual tonsil is poorly developed and cannot be observed macroscopically [1].

Palatine tonsils (*tonsilla palatina*) are lymphoid organs contained in the tonsillar fossa; this is bounded dorsally via the soft palate, and ventrally by the root of the tongue. These structures are located within the oropharynx (*pars oralis pharyngis*) and extended from the isthmus of the fauces (*isthmus faucium*) to the base of the epiglottis. On each side, there is the palatoglossal arch (*arcus palatoglossus*), located ventrally to the tongue, and dorsally to the soft palate. Positioned laterally to the palatoglossal arch is the pterygomandibular fold (*plica pterygomandibularis*).

The reddish palatine tonsil is proportionally larger in the cat compared to the dog, since the semilunar fold also contains lymphoid tissue mainly composed of secondary lymphoid follicles and inter-follicular regions [1,2,3,4].

The palatine tonsil, hidden in the tonsillar fossa, is a long and relatively thin lymphoid organ, with an oval shape on a transverse section; it is divided into a protruding fusiform portion, the major portion of the organ, and a usually smaller and deeper minor portion that lies deep to the mucosa, forming the rostral part of the lateral wall of the tonsillar fossa. The deep portion of the gland may be formed as a result of some insult; it is usually absent in young, healthy animals [1,2,3,4]. 

Tonsils are set in the buccopharyngeal fascia; laterally to this lies the lingual branch of the glossopharyngeal nerve and the styloglossal and medial pterygoid muscles.

The palatine tonsil has no lymphatic afferents. Its efferent vessels drain into the medial retropharyngeal lymph nodes.

They consist of aggregations of lymphoid cells. Its location at the crossing of the digestive and respiratory tracts plays a key role in immunity, as this is the site where vast amounts of foreign antigens enter the body during feeding and breathing [1,2,3,4]. 

MRI is considered the technique of choice in depicting soft tissue, and it is also able to show possible concurrent bone involvement or invasion [5,6]. In human medicine, MRI is commonly used in the investigation of tonsillar diseases, including neoplasia [7], and increasing interest has been also given to take a first step towards high spatial resolution 3D imaging of the human palatine tonsil [8]. In veterinary medicine, a recent study conducted on healthy dogs has described the MRI appearance of presumed normal canine palatine tonsils [6]. Canine tonsils were located at the level of the temporomandibular joints. The majority were rounded to oval with smooth and well-defined borders. All tonsils were hyperintense to the medial pterygoid muscles in T1-weighted, T2-weighted, FLAIR, and T2* gradient echo images, and they showed either homogeneous or heterogeneous signal intensity, with marked or moderate contrast enhancement [6].

This study aimed to evaluate the feasibility of low-field MRI to detect palatine tonsils in cats, and to describe the MRI features of presumed normal palatine tonsils, assessing the interoperator reproducibility of the findings.

## 2. Materials and Methods

This is an anatomic, observational, and retrospective study. MRI studies of the heads of cats were reviewed. Cats who underwent MRI for investigation of neurological signs at the University Veterinary Teaching Hospital of Teramo in 2022 were reviewed. 

The inclusion criteria included the following: cats at least 1-year-old at the time of the image acquisition, since tonsils in young animals are physiologically prominent due to the hyperplasia of the lymphatic tissue during the ‘maturation’ of the immune system and protrude from the epithelial pocket [5,9,10]. No clinical signs of disease in the area of drainage of the tonsils, including coughing, sneezing, nasal discharge, vomiting, or swelling of the head and neck, or if they had the suspicion of both oral or proximal airway inflammatory processes or neoplasia. 

The cats included had normal MRI studies or intracranial disease (both inflammatory or neoplastic); the exclusion criteria were: primary malignancy or inflammatory processes affecting the oral or nasopharyngeal tissues, or primary malignancies known to metastasize to palatine tonsils. Images were acquired with a low-field magnetic resonance (Esaote 0,25 T VET-MR Grande—Esaote Genova, Italy).

The cats were premedicated with 0.2 mg/Kg methadone (Semfortan 10mg/mL, Dechra Eurovet Animal Health B.V.—Netherland) and 4 mcg/Kg dexmedetomidine (Dexdomitor 0.5 mg/mL Vetoquinol, Italy). Anesthesia was induced via the intravenous administration of 4 mg/Kg propofol (PropoVet 10mg/mL Zoetis Roma, Italy), which, after endotracheal intubation, was maintained via the administration of isoflurane (Vetflurane, Virbac SA, Carros, France) and oxygen.

All the MRI studies included were performed with the patient under general anesthesia and in sternal recumbency. For each patient, multiple pulse sequences and image planes were available, including T2-weighted (T2W) images in at least the sagittal and transverse and transverse T1-weighted (T1W) acquired pre- and post-contrast (Prohance, Gadoteridolum, 0.5 mmol/mL, Bracco, Milan, Italy), FLAIR, and T2* gradient echo images. Post-contrast images were obtained 1 to 1.5 min after intravenous contrast medium administration. 

The sagittal sequences were aligned parallel to the midline of the brain, with a slice thickness of 3 mm and an inter-slice interval of 0.3 mm. 

The transverse sequences were aligned perpendicular to the hard palate in all studies, with a slice thickness ranging from 3 to 4 mm and an inter-slice interval ranging from 0.3 to 0.4 mm. 

The dorsal sequence, when available, was parallel to the hard palate, with a slice thickness of 4 mm and an inter-slice interval of 0.4 mm.

The images were reviewed and analyzed using an open-source DICOM viewer software (Horos project); both observers evaluated the MRI study, knowing that the cats included could have a normal study, inflammatory intracranial disease, or neoplastic intracranial disease. Qualitative and quantitative features were evaluated by an ECVDI II-year resident and a veterinary researcher with more than 5 years of experience in MRI. 

Each observer optimized the window level and window width according to their discretion.

At first, the observers evaluated whether the palatine tonsils were visible or not, considering the temporomandibular joint (TMJ) as an anatomical landmark based on the previously published literature [6]. 

Each observer assigned a quantitative score based on the structure’s visibility (0, 1, or 2): zero if the tonsil was considered not visible, one if the tonsil was considered barely visible, and two if the tonsil was considered clearly visible. 

From the qualitative analysis, the margins of the tonsils were classified as well-defined or ill-defined in the T2W transverse images.

The cross-sectional shape of the tonsils was subjectively defined as rounded, oval, or elongated in the T2W transverse images.

The signal intensity of the tonsils was subjectively classified as hyperintense, isointense, or hypointense compared with the medial pterygoid muscles. When the signal was heterogeneous, the authors tried to detect particular patterns depending on the hyperintensity distribution in the tonsils (either centrally or peripherally). Contrast enhancement was subjectively evaluated in post-contrast T1W transverse images. If enhancement was present, it was classified for its distribution. The degree of enhancement was defined as mild, moderate, or marked. For the quantitative assessments, the cross-section and tonsil’s volume were measured using an electronic caliper on the transverse plane of the T2w images, based on the results of the available literature [6]. 

The quantitative data were measured by each author. Concerning the statistical analysis, data regarding the short/long axis and volume were expressed as mean +-standard deviation, with normal distribution determined with the Shapiro–Wilk test.

The agreement between the operators was assessed through Pearson’s coefficient for the quantitative evaluations, considering a high agreement with a coefficient between +0.5 and +1, and through the Chi2 test for the other qualitative parameters, with a *p* < 0.05 set as a statistically significant difference. 

A direct comparison was performed on tonsils observed by both operators in the same cat.

All statistical analyses were performed with Past4 software.

## 3. Results

Fourteen domestic short hair cats met the inclusion criteria and were included in this study. There were four female cats and ten male cats; the female cats were all spayed, and there were nine neutered male cats and one intact male (mean age: 7.2 years ± 2.3 years, meant weight: 3.5 ± 1.2 kg). 

All patients were admitted to our facility due to exhibiting neurological signs (e.g., seizures, head tilt, or behavioral changes), and six cats were strays and polytraumatized with spinal trauma and concurrent abnormal mentation.

All MRI studies were performed for the evaluation of the brain.

TMJs were used as a consistent landmark to detect the feline palatine tonsils in the transverse images of this cohort of cats. In the transverse images, the tonsils were located in the lateral walls of the oropharynx extending rostrally and caudally to the TMJ. Both observers described a consistent location of the tonsil in the slice immediately caudal to the TMJ.

Although the tonsils were not visible in all the patients, sufficient data were collected to outline their main features. The first author observed an overall n = 12/14 right tonsils and n = 8/14 left tonsils (overall n = 20/28 tonsils; 71%), while the second author observed an overall n = 11/14 right tonsils and n = 9/14 left tonsils (overall n = 20/28 tonsils; 71%). 

**Figure 1:**
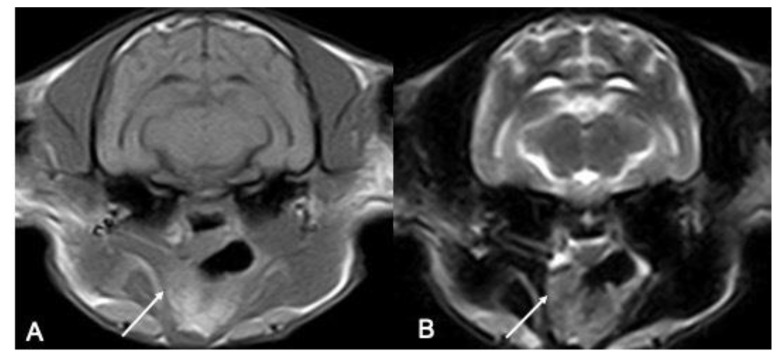
(**A**,**B**) **Comparison between the right tonsil and left tonsil in a cat.** In this picture, there is an example of a cat where the right tonsil is pointed with by the solid white arrow, but its contralateral part is not evident.

The right tonsil was more consistently visualized than the right, with a good agreement between the operators, except for one left tonsil detected only by one of the two observers (Table 1) (Figure 1).

**Figure 2:**
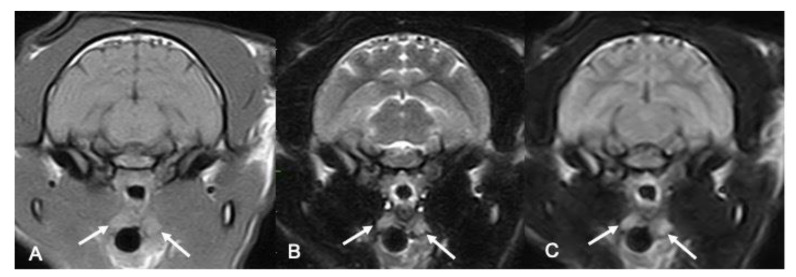
**MRI features on feline tonsils.** In this picture, both the left and right tonsils are pointed with the solid white arrows in the T1w sequence (**A**), T2w sequence (**B**), and FLAIR sequence (**C**). In all the panels, the tonsils appear as ovoid, well-defined structures that are hyperintense compared to the medial pterygoid muscle, with an homogeneous appearance in T1w and FLAIR, and with a thin hyperintense peripheric rim in T2w.

Where visible, the tonsils’ margins were classified as both ill-defined and well-defined for both the right side (n = 5 for O1 and n = 4 for O2 well-defined; n = 7 for O1 and n = 8 for O2 ill-defined) and the left side (n = 3 for O1 and O2 well-defined; n = 4 for O1 and O2 ill-defined), with no difference between the operators (Table 2) (Figure 2).

The shape was more often described as oval (n = 8 for O1 and n = 7 for O2 for the right one, and n = 5 for O1 and n = 3 for O2 for the left one, respectively), with no significant difference between the operators (Table 2) (Figure 2).

Regarding the size of the tonsils, the measurements are resumed in Table 3; despite no difference being observed between the mean values for the short axis, a negative correlation was observed between the operators, while a moderate-to-good agreement was observed for the long axis and volume.

Concerning the signal, the majority of the tonsils were defined as hyperintense in the T2W (n = 13/20 tonsils both on the right and the left) and T1W sequences (11/20 right tonsils and 7/14 left tonsils). Both the right and left tonsils appeared as hyperintense on the FLAIR sequences (Figure 2).

**Figure 3:**
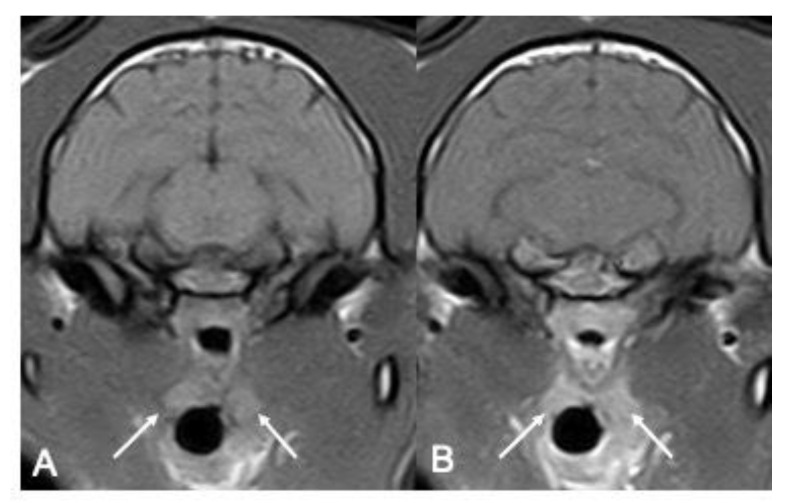
Pre- and post-contrast T1w images of feline tonsils. In panel (**A**), pre-contrast T1w images of the tonsils are pointed with solid white arrows; in panel (**B**), note the intense and homogeneous contrast enhancement.

The main disagreement between the two authors affected the description of the T1W signal for the right tonsils (*p* = 0,04), while for the left tonsil, there was mild agreement (*p* = 0,13). In the T2W sequences, the most consistent appearance of tonsils comprised both the presence of a T2W hyperintense peripheral signal and a homogenous T2 hyperintense signal for both the right and left tonsils. All the tonsils included showed contrast enhancement in the T1W post-contrast sequence; both right and left tonsils displayed a diffuse heterogeneous or homogeneous contrast enhancement (Table 4) (Figure 3). In only one patient, one observer described the presence of a signal void rim in the T2*W sequence. 

## 4. Discussion

In this study, palatine tonsils in cats were retrospectively assessed in low-field MRI studies of the head by two different observers.

For each tonsil on the right and left side, the visualization, shape, margins, size, signal intensity, and pattern were recorded and compared between the two observers.

The palatine tonsils in this study were located at the level of the TMJs, extending caudally from this anatomical landmark. This result is slightly different compared with the results that have been obtained on canine species, in which their tonsils had their largest transverse cross-section at the level of the temporomandibular joints. Therefore, TMJs can be used as a consistent landmark for the evaluation of feline tonsils. In this study, the feline palatine tonsils were mostly described as ill-defined with an oval shape. These results differ from the canine ones, as tonsils in dogs were mostly assessed as well-defined with variable round to ovoid shapes [6]. These differences could be due to the different kinds of magnets used. The canine-cited study was performed with a high-field magnet (1.5 Tesla), and our examinations were all performed with a low-field machine (0.25 Tesla) that allowed for a good assessment of the tonsils. In the authors’ opinion, the other variable to take into account is the size of the included subjects, weighing up to 4.5 kg. Our population was homogeneous and comprised small patients, compared with the dogs described by Ruiz-Debring (2018) [6], in which a wide variety of different breeds and body weights were present. The best visualization of the tonsils is desirable in larger patients. Our population was more homogeneous concerning the canine one, and this makes the results most consistent. Further studies with a high-field magnet are desirable for a better evaluation and comparison. The authors found some discrepancies between the evaluation of the long axis and the assessment of the left tonsils compared to the right ones. 

The authors tried to explain this difference in their results, referring to the appearance of the margins. There was a slight prevalence of ill-defined margins.

Furthermore, the authors tried to interpret both of these results by referring to the following other factors: All the studies were performed for the evaluation of the brain, and therefore the positioning of the animals was conducted to obtain symmetric images of the brain structures. Moreover, the tonsils are soft tissue anatomic structures, which, during positioning, could be affected by the pressure exerted by nearby structures, and this could modify their shape and position. It is desirable if the MRI study is performed to evaluate the tonsils using oblique scans, one on each side, or to perform 3D sequences, which, in the absence of inter-slice gaps, allow for a more precise spatial evaluation, especially of very small anatomical structures [11,12]. The thickness of the slices in our study ranged from 3 to 4 mm, with an interslice gap of 10% of the slice thickness; since the tonsils are millimetric anatomical structures (the largest dimension found was 5.5 mm), it is easy to lose information with a non-targeted setting of the slice. 

The authors did not find any reference describing a mild difference in the localization of both tonsils; subsequently, they hypothesized the presence of a physiological asymmetry in the localization of the tonsils, as it happens with other paired organs within the body.

The rim-signal void described by an observer within the bilateral tonsils of a cat could be explained by referring to the history of that patient; that cat was a stray and polytraumatized, so that kind of signal could be due to the presence of a hemorrhage. We believe that this signal is less likely attributable to the presence of gas within the tonsil’s parenchyma, due to the symmetrical distribution described by the author in the two tonsils.

The cats included were presumably not affected by pathologies involving the palatine tonsils; certainly, the evaluation of a pathological tonsil should be easier for the increased volume and possible changes in their shape and appearance. Common tonsillar diseases include inflammatory and primary neoplastic processes. Feline oral squamous cell carcinoma (SCC) is the most common oral neoplasia in cats [13], and the second most common in dogs [14]. The most common locations of oral SCC in cats are the sublingual/lingual region, maxilla, mandible, buccal mucosa, lip, and caudal pharynx/tonsillar region. Historically, feline SCC has been considered to have a low metastatic rate, with metastasis to regional lymph nodes or, even more rarely, to the lungs [15]. Oral fibrosarcoma is the second most common oral tumor in cats and is unlikely to metastasize. Lymphoma can also affect the tonsils bilaterally and is usually accompanied by generalized lymphadenopathy. Other cancers, especially malignant melanoma, can metastasize to the tonsils [14]. Cervical lymphadenopathy is a common presenting sign of tonsil pathology, even with very small primary tonsillar cancers. 

In human medicine, MRI is commonly used in the investigation of tonsillar disease, including oral-oropharyngeal squamous cell carcinoma and nasopharyngeal carcinoma [7,15].

This study has some limitations; the first one is the small population of cats included, which is related to the strict inclusion criteria the authors used; at first, feline patients were included if they were at least 1-year-old and then, to describe normal tonsils, all the cats with diseases affecting their oral or nasal cavities were excluded. The second limitation is the low-field magnet used; however, the authors have proven the assessability of feline tonsils even with a low-field magnet. Therefore, the use of a high-field magnetic resonance can only make this evaluation better. 

The third limitation is the lack of a standardized protocol for the MRI studies; moreover, to the authors’ knowledge, there are no studies describing an MRI protocol for the evaluation of feline tonsils, and it is desirable to develop one on the basis of the capability to evaluate tonsils with this modality. The fourth limitation is the absence of cytological or histological confirmation of the lack of any pathologies affecting the included tonsils. This is related to the retrospective nature of the study, but the absence of cervical lymphadenopathy and any other gross abnormality in the upper airways and oral cavity makes the possibility of the presence of a disease poor. 

The last limitation is related to the methodology used. MRI is a non-invasive imaging technology that yields three-dimensional detailed anatomical images; however, this is a time-consuming procedure, and it is expensive. For these reasons, other faster and less expensive methods may be preferred. CT, although similarly expensive, could overcome the problem of long MRI execution times, while remaining a cross-sectional method. A previous study has reported the availability of CT to examine palatine tonsillar neoplasia in dogs and has emphasized the importance of evaluating the lymph nodes in the assessment of tonsil pathologies and, therefore, of using cross-sectional methods that also allow the tissues close to the tonsils to be investigated [16].

Ultrasound, which is certainly more accessible from an economic point of view, does not allow for a cross-sectional evaluation of the neck and tonsils; however, it was reported to be comparable and complementary to CT and MRI as a safe alternative in human medicine [17,18,19,20,21,22,23]. This procedure is accessible and much less expensive, and it is reliable to obtain measures of the palatine tonsils [24,25], especially in children, without exposing the patient to ionizing radiations.

## 5. Conclusions

In conclusion, this study has shown that MRI is a potentially useful imaging modality for the assessment of palatine tonsils in cats, also with a low-field magnet, and their normal MRI appearance has been described for the first time. 

On the basis of our results, we recommend the evaluation of tonsils in the transverse plane, and we consider it most useful and accurate for the estimation of the short axis, at least when this evaluation is made on MRI studies designed to evaluate the brain, suggesting the use of 3D sequences to improve the visualization of the tonsils where this is not achievable with standard sequences. This study could be used as a baseline for the investigation of the value of MRI in the assessment of tonsils with high-field MRI and for tonsillar disease in cats.

## Figures and Tables

**Table 1:** **Visualized tonsils recorded by each observer.** In this table, the tonsils visualized by each operator have been recorded for both the left side and the right side; the p value > 0.05 points that there is no difference between the observers (O1 and O2); the test used was the Chi2 test.

	Right		Left	
	**O1**	**O2**	**O1**	**O2**
**Not visible**	n = 2	n = 2	n = 6	n = 5
**Barely visible**	n = 7	n = 4	n = 5	n = 6
**Clearly visible**	n = 5	n = 7	n = 3	n = 3
***p*** **-Value**	0.74		0.7	

**Table 2:** **Classification of the shape and margins of the tonsils recorded by the observers.** In this table, the data about shape and margins classification are reported by both of the observers; the *p*-value > 0.05 points that there is no difference between the observers (O1 and O2); the test used was the Chi2 test.

Shape	Right		Left		Margin	Right		Left	
	O1	O2	O1	O2		O1	O2	O1	O2
**Oval**	n = 8	n = 7	n = 5	n = 3	**Well defined**	n = 5	n = 4	n = 3	n = 3
**Elongated**	n = 4	n = 3	n = 1	n = 1	**Ill defined**	n = 7	n = 8	n = 4	n = 4
**Round**	n = 0	n = 1	n = 2	n = 3					
***p*** **-Value**	0.38		0.47			0.67		0.75	

**Table 3:** **Quantitative assessment of the short axis, long axis, and volume.** In this table, data collected by both observers (O1 and O2) are recorded; the negative Pearsons coefficient points the lack of correlation between the two observers for the long axis, while for the short axis and volume, the correlation is moderate-to-good. The long and short axes are expressed in millimeters (mm), with the volume in mm^3^. The values reported are the mean and standard deviation (st.dev). The test used was the Pearson’s coefficient test.

	**Right**					
	**Long axis (mm)**		**Short axis (mm)**		**Volume (mm^3^)**	
	**O1**	**O2**	**O1**	**O2**	**O1**	**O2**
**Mean ± st.dev**	3.91 ± 0.85	4.63 ± 0.7	2.10 ± 0.74	2.91 ± 1.1	8.04 ± 1.93	13.6 ± 6.1
**Pearson’s coefficient**	−0.26		0.46		0.5	
	**Left**					
	**Long axis (mm)**		**Short axis (mm)**		**Volume (mm^3^)**	
	**O1**	**O2**	**O1**	**O2**	**O1**	**O2**
**Mean ± st.dev**	3.7 ± 0.8	4.86 ± 0.69	2.20 ± 0.52	3.36 ± 0.58	7.98 ± 1.99	16.5 ± 4.27
**Pearson’s coefficient**	−0.47		0.8		0.37	

**Table 4:** **Signal intensities and patterns of the tonsils.** In this table, both the signal intensity and pattern distribution have been reported by each observer (O1 and O2). The *p*-value > 0.05 points that there is no difference between the observers, except for a slight difference for the T1W signal pattern. The test used was the Chi2 test.

**Signal intensity**	**T1W right**		**T1W left**		**T2W right**		**T2W left**		**FLAIR right**		**FLAIR left**		**T1W post right**		**T1W post left**	
	**O1**	**O2**	**O1**	**O2**	**O1**	**O2**	**O1**	**O2**	**O1**	**O2**	**O1**	**O2**	**O1**	**O2**	**O1**	**O2**
**Hyperintense**	n = 6	n = 5	n = 3	n = 4	n = 12	n = 10	n = 7	n = 7	n = 12	n = 12	n = 7	n = 7	n = 10	n = 10	n = 7	n = 7
**Iso-iperintense**	n = 2	n = 4	n = 2	n = 2	n = 0	n = 1	n = 0	n = 0	n = 0	n = 0	n = 0	n = 0	n = 2	n = 0	n = 0	n = 0
**Isointense**	n = 4	n = 3	n = 2	n = 1	n = 0	n = 1	n = 0	n = 0	n = 0	n = 0	n = 0	n = 0	n = 0	n = 2	n = 0	n = 0
***p*-Value**	**0.63**		**0.34**		**0.33**		**1**		**1**		**1**		**0.36**		**1**	
**Signal pattern**	**T1W Right**		**T1W left**		**T2W Right**		**T2W left**		**FLAIR right**		**FLAIR left**		**T1W post right**		**T1W post left**	
	**O1**	**O2**	**O1**	**O2**	**O1**	**O2**	**O1**	**O2**	**O1**	**O2**	**O1**	**O2**	**O1**	**O2**	**O1**	**O2**
**Homogeneous**	n = 9	n = 5	n = 5	n = 2	n = 7	n = 4	n = 3	n = 4	n = 3	n = 4	n = 4	n = 4	n = 3	n = 3	n = 2	n = 3
**Peripherical hyperintens rim**	n = 3	n = 2	n = 2	n = 1	n = 3	n = 6	n = 5	n = 2	n = 3	n = 2	n = 1	n = 1	n = 4	n = 4	n = 2	n = 2
**Heterogeneous**	n = 0	n = 5	n = 0	n = 3	n = 2	n = 2	n = 0	n = 0	n = 3	n = 3	n = 2	n = 2	n = 0	n = 1	n = 3	n = 2
***p*-Value**	**0.04**		**0.13**		**0.40**		**0.22**		**1**		**1**		**0.76**		**0.29**	

## Data Availability

All the data available are included in the main manuscript.
